# Metabolomics analysis of gut barrier dysfunction in a trauma-hemorrhagic shock rat model

**DOI:** 10.1042/BSR20181215

**Published:** 2019-01-08

**Authors:** Zhongqi Li, Jian Li, Shouwei Zhang, Gang Chen, Shaohua Chi, Xugang Li, Fei Guo, Jianbo Zhu, Baoxi Sun

**Affiliations:** 1 Department of Critical Care Medicine, Rizhao People’s Hospital, Rizhao, Shandong, 276800, China; 2 Stroke Center, Rizhao Central Hospital, Rizhao, Shandong, 276800, China; 3 Department of Respiratory, Rizhao People’s Hospital, Rizhao, Shandong, 276800, China; 4 Department of Vascular Interventional, Binzhou Medical University Hospital, Binzhou, Shandong, 256603, China; 5 Department of Respiratory, Yantai affiliated Hospital of Binzhou Medical University, Yantai, Shandong, 264100, China; 6 Department of Respiratory, Binzhou People’s Hospital, Binzhou, Shandong, 256600, China; 7 Department of Critical Care Medicine, Affiliated Caner Hospital of Zhengzhou University and Henan Cancer Hospital, Zhengzhou, Henan, 450008, China

**Keywords:** Gut barrier, Metabolism, Metabolites, Metabolomics, Trauma-hemorrhagic shock

## Abstract

Intestinal barrier dysfunction has been implicated in the development of multiorgan dysfunction syndrome caused by the trauma-hemorrhagic shock (THS). However, the mechanisms underlying THS-induced gut barrier injury are still poorly understood. In the present study, we used the metabolomics analysis to test the hypothesis that altered metabolites might be related to the development of THS-induced barrier dysfunction in the large intestine. Under the induction of THS, gut barrier failure was characterized by injury of permeability and mucus layer, which were companied by the decreased expression of zonula occludens-1 in the colon and increased levels of inflammatory factors including tumor necrosis factor-α, interferon-γ, interleukin (IL)-6, and IL-1β in the serum. A total of 16 differential metabolites were identified in colonic tissues from THS-treated rats compared with control rats. These altered metabolites included dihydroxy acetone phosphate, ribose-5-phosphate, fructose, glyceric acid, succinic acid, and adenosine, which are critical intermediates or end products that are involved in pentose phosphate pathway, glycolysis, and tricarboxylic acid cycle as well as mitochondrial adenosine triphosphate biosynthesis. These findings may offer important insight into the metabolic alterations in THS-treated gut injury, which will be helpful for developing effective metabolites-based strategies to prevent THS-induced gut barrier dysfunction.

## Introduction

Trauma is the leading cause of death for those under 45 years of age in the United States, and the trauma-hemorrhagic shock (THS) is a severe disorder and most frequent cause of mortality that is caused by massive tissue injury [1]. Studies focusing on the pathogenesis of THS demonstrate that it causes the injury of many distant organs via the so-called multiorgan dysfunction syndrome (MODS), which is thought to be caused, at least in part, by excessive activation of systemic inflammatory responses [2]. The intestinal tract contains large amounts of bacteria and the translocation of bacteria or other microbial molecules from the intestine to the systemic circulation has been considered as an important contributor for the development of systemic inflammation [3]. Therefore, it is gradually recognized that intestinal injury and the subsequent loss of barrier function have been implicated in the development and the initiation of THS-induced MODS [4]. Therefore, prevention or amelioration of intestinal barrier dysfunction would be a key therapeutic strategy for the prevention of THS-associated MODS.

Intestinal barrier function, which is composed of the intestinal epithelium, is considered to be a crucial mediator for the maintenance of intestinal homeostasis [5]. Intestinal mucus layer provides a barrier protecting the epithelium from the invasion and infection of pathogens [6]. The pathogenesis of THS-induced intestinal injury and barrier failure have been reported by some studies. For instance, reactive oxygen species-mediated mucus layer damage might play an important role in contributing THS-induced intestinal barrier injury [7,8]. Further study demonstrated that the interaction of luminal digestive enzymes and intestinal mast cells might contribute to the protective role of mucus layer on THS-induced MODS [9]. Moreover, the activation of toll-like receptor-4 in the intestinal epithelium was also required for the induction of endoplasmic reticulum stress and release of circulating HMGB1 during the development of THS-induced acute lung injury [10]. However, these findings are not enough to elucidate the pathogenesis of THS-induced intestinal barrier function, so much work is still required to understand the detailed mechanisms.

A key hallmark of major traumatic injury is abnormal metabolic changes in tissues and organs, which is caused by inadequate supply of systemic oxygen and nutrients [11]. Catabolism, acidosis, and insulin resistance with resultant hyperglycemia have been considered as the metabolic phenotype for THS-induced secondary injury [12]. Abnormal metabolic changes might also be related to the systemic inflammatory responses [13]. Since the degree of metabolic acidosis has been demonstrated to predict the severity of acute lung injury in trauma patients, understanding the critical role of metabolic changes in response of THS is of great significance [14]. Metabolomics is a growing field of systemic biology that can acquire an overview of the metabolic changes in a given biological system by quantitatively measuring many small-molecule metabolites [15]. Indeed, studies have demonstrated that a number of metabolites were changed in the plasma of severely injured trauma patients and THS rat model [16,17]. However, the metabolic changes in the colonic tissue under the induction of THS have not yet been investigated.

In the present study, a non-targeted metabolomics approach based on gas chromatography coupled to mass spectrometry (GC/MS) in conjunction with univariate and multivariate statistical analyses was performed to comprehensively determine the metabolic alterations of THS-induced gut injury, with the aim of identifying potential small-molecule metabolites contributing to the pathophysiology of THS-induced gut barrier failure. To best of our knowledge, this is the first study to apply metabolomics analyses to examine the metabolic mechanisms underlying THS-induced intestinal barrier dysfunction.

## Materials and methods

### Animal model

A total of 20 Sprague-Dawley rats weighing 350–500 g were maintained in barrier-sustained conditions with 12-h light–dark cycles and allowed free access to food and water before use. According to a table of ‘random numbers’, rats were randomly divided into two groups (*n*=10) including the THS group and the control group. The animal experiments were performed in accordance with the guidelines of the local ethics committee.

THS is performed as previously described [18,19]. Briefly, rats were anesthetized with 50 mg/kg pentobarbital sodium via intraperitoneal injection. Soft tissue trauma was performed with midline laparotomy and then the femoral artery and vein were cannulated. The rats were allowed to awaken, after which they were bled within 10 min to reach the mean arterial pressure of 30–35 mm Hg at a rate of 1 ml per min and maintained for 90 min. The mean arterial pressure was monitored using a ProPaq invasive monitoring device. The rats are resuscitated with their shed blood at a rate of 1 ml per min and observed for 3 h. The rats that only received trauma were defined as the control group.

### Assessment of intestinal permeability

Intestinal permeability was assessed *in vivo* using the fluorescein isothiocyanate (FITC)-labeled dextran according to the method described previously [20]. Briefly, after the 3-h fasting period was completed, rats were orally administrated with FITC-labeled dextran. Blood was collected 5 h later and was then centrifuged at 1000 rpm for 20 min to separate serum. Fluorescence intensity in the serum was determined at 485-nm excitation and 520-nm emission wavelengths.

### Tissue collection and colon histology

Rats were anesthetized with CO_2_ inhalation 3 h after the THS induction and followed by cervical dislocation. The colonic section was dissected, and a 5-mm segment of distal colon was fixed in freshly prepared 4% (w/v) paraformaldehyde (pH 7.0), processed and embedded in paraffin. Colon pieces from rats were sectioned and stained with hematoxylin and eosin (H&E) staining for histological examination.

### Mucus production measurement

Paraffin-embedded distal colonic tissues were sectioned at 5 μm thickness, deparaffinized and subjected to alcian blue (AB) staining for mucus content measurement.

### Immunohistochemistry

Following deparaffinization and rehydration, colonic sections were blocked with 5% bovine serum albumin for 30 min at room temperature and then washed with phosphate-buffered saline (PBS). Tissue sections were incubated with primary antibody zonula occludens (ZO)-1 (Invitrogen, Eugene, OR, U.S.A.) overnight at 4°C. Slides were washed three times in PBS before applying peroxidase-conjugated secondary antibody for 2 h at room temperature.

### Cytokine analysis

Cytokine levels of tumor necrosis factor (TNF)-α, interferon (IFN)-γ, interleukin (IL)-6, and IL-1β levels in the serum were examined by ELISA kit (Nanjing Jiancheng, Nanjing, China) according to the manufacturer’s instructions.

### GC/MS analysis

GC/MS-based metabolomics was performed by ProfLeader Biotech Co, Ltd (Shanghai, China) according to the methods described in previous publication with some minor modifications [21]. In brief, each colonic sample mixed with water was vortexed prior to centrifugation. The supernatant was transferred to a GC vial containing internal standards. The mixture was dried under gentle nitrogen stream and then added with methoxyamine hydrochloride in pyridine. The resultant mixture was vortexed vigorously and incubated at 37°C for 90 min. Derivatization was performed by adding BSTFA (with 1% TMCS) into the mixture. The derivatized samples were analyzed by an Agilent 7890A gas chromatography system coupled to an Agilent 5975C inert MSD system (Agilent Technologies Inc., CA, U.S.A.). An HP-5MS fused-silica capillary column was utilized to separate the derivatives. Helium was used as a carrier gas at a constant flow rate through the column. The samples were analyzed in a random sequence.

### Data preprocessing and identification of metabolites

The acquired GC/MS data were imported to SIMCA Statistical Analysis (version 13.0, Umetrics AB, Umeå, Sweden), where multivariate statistical analyses including partial least-squares discriminant analysis (PLS-DA) and orthogonal partial least-squares discriminant analysis (OPLS-DA) were performed [21]. The differential metabolites were determined by the combination of the Variable importance in the projection (VIP) value (>1) of PLS-DA model and the *P*-values (<0.05) from two-tailed Student’s *t*-test on the normalized peak intensities. Fold change should be calculated as the ratio of average normalized peak area between the two groups. The structural identification of differential metabolites was performed by AMDIS software, where the purified mass spectra were automatically matched with an in-house standard library including retention time and mass spectra, Agilent Fiehn GC/MS Metabolomics RTL library and Golm Metabolome Database, respectively.

### Statistical analyses

Prism 6.0 (GraphPad Software, San Diego, CA) was used for the statistical analyses. Comparisons for animal experiments between two groups were analyzed using the non-parametric Mann–Whitney test. Statistical significance was defined as *P*-value of 0.05.

## Results

### The gut barrier function is impaired in THS-treated rats

**Figure 1:**
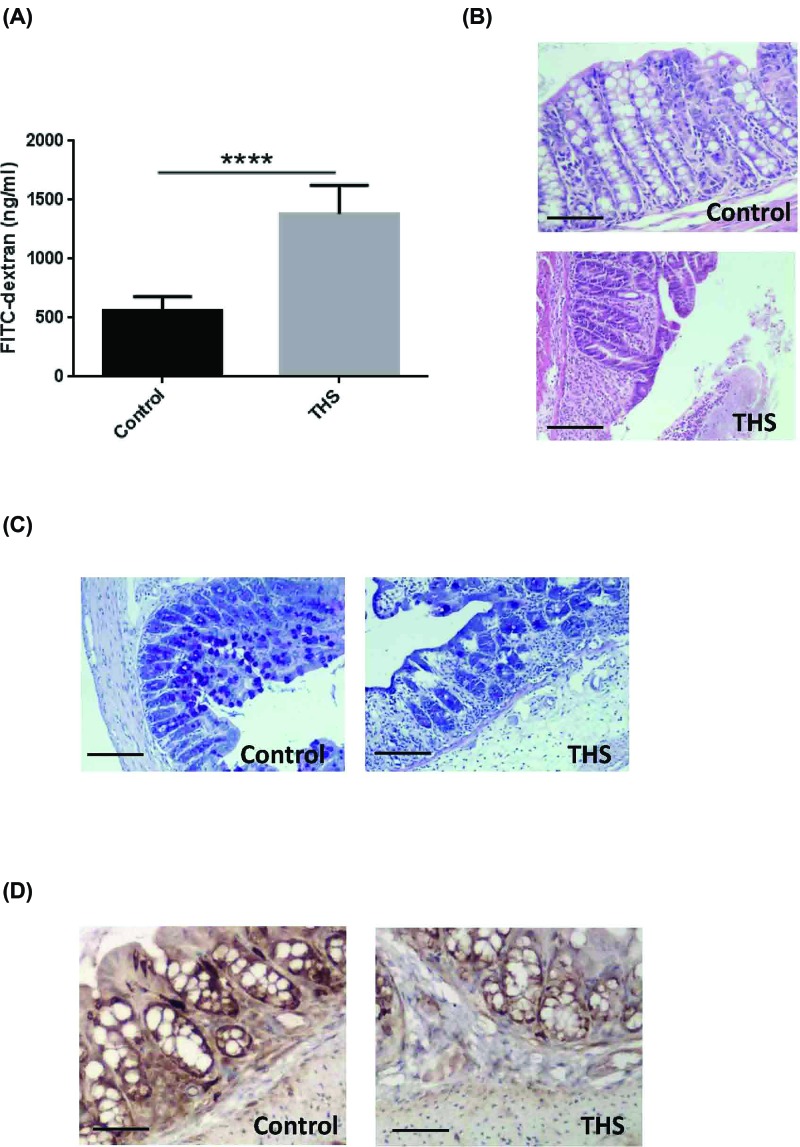
THS induces intestinal barrier dysfunction in the colon of rats (**A**) Intestinal permeability was determined by orally administration with FITC-labeled dextran. (**B**) Representative images of the colonic tissues analyzed by H&E staining. Scale bar = 100 μm. (**C**) Representative images of the colonic tissue analyzed by AB staining. Scale bar = 100 μm. (**D**) Immunohistochemistry analysis of expression level of ZO-1. Scale bar = 100 μm. The data are expressed as means ± SEM. **** *P*<0.0001 by Mann–Whitney test.

We first confirmed that THS could induce intestinal barrier dysfunction by using a THS-treated rat model. Following the induction of THS, intestinal permeability was examined by using the method of FITC-labeled dextran. Serum level of FITC-dextran was significantly elevated in THS-induced rats compared with that of control rats (Figure 1A). The histological evaluation of colonic tissue from control rats revealed a normal structure without histological changes. In contrast, THS treatment induced serious injuries to the colon of rats. Most of the epithelial cells were disappeared along with the loss of mucosa and crypts and marked infiltration of granulocytes and mononuclear cells into the mucosa and submucosa (Figure 1B). Consistently, the number of mucus-producing crypt cells was also significantly decreased in the colon of THS-induced rats compared with that of control rats, suggesting that THS could cause loss of mucus layer (Figure 1C). Immunohistochemistry analysis suggested that the expression level of the tight junction protein ZO-1 was significantly decreased in THS-treated rats compared with that of control rats (Figure 1D). Collectively, these data suggest that THS could induce impairment of the gut barrier integrity and loss of the mucus layer.

### The excessive inflammatory factors are induced in THS-treated rats

**Figure 2:**
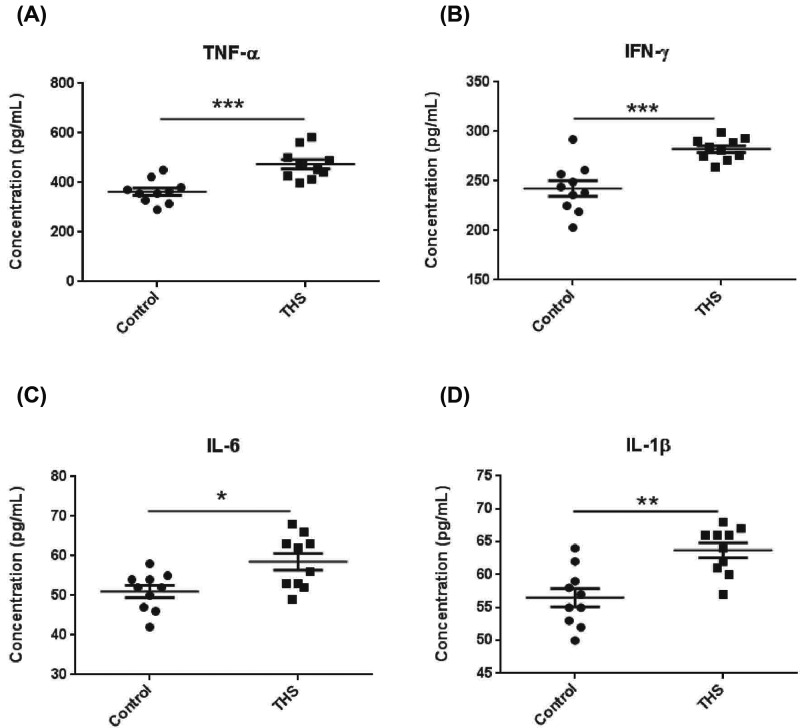
THS promotes production of inflammatory factors in the serum of rats (**A**–**D**) Inflammatory mediator levels of TNF-α, IFN-γ, IL-6, and IL-1β in serum were examined by ELISA. The data are expressed as means ± SEM. *P<0.05, ** *P*<0.01, and *** *P*<0.001 by Mann–Whitney test.

Gut barrier dysfunction is an important contributor for the development of systemic inflammatory responses [3]. To investigate whether THS-induced intestinal barrier failure could cause excessive systemic immune responses, we detected the serum levels of inflammatory cytokines including TNF-α, IFN-γ, IL-6, and IL-1β by using ELISA. THS induced significantly increased production of TNF-α, IFN-γ, IL-6, and IL-1β compared with that of control rats (Figure 2A–D). Thus, these results suggest that THS-induced intestinal barrier function might cause severe pro-inflammatory status in the circulation system.

### Colonic metabolome is changed under the induction of THS

**Figure 3:**
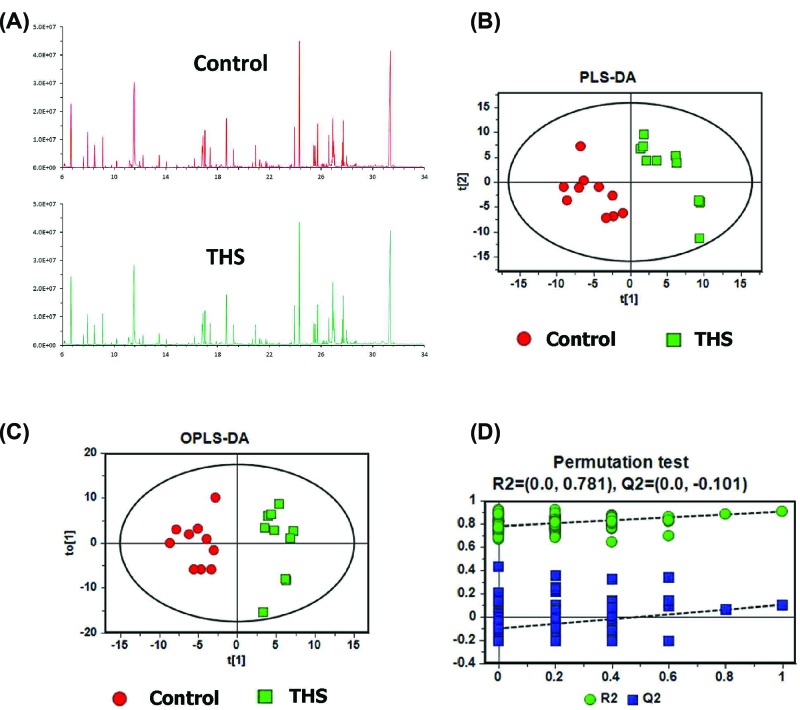
THS induces different metabolomic profiles in colonic tissues (**A**) Representative GC/MS TIC chromatograms in colonic tissue of rats from control and THS groups. (**B**) PLS-DA, (**C**) OPLS-DA, and (**D**) permutation test of PLS-DA for the metabolomic profiles in colonic tissue of rats from control and THS groups.

GC/MS-based metabolomics was conducted to profile the colonic metabolome of THS and control rats. Representative total ion current (TIC) chromatographs from the two groups are shown in Figure 3A, respectively. As a supervised multivariate statistical model, PLS-DA model was performed to illustrate the metabolic differences between THS and control rats. In PLS-DA score plots, each plot represents a sample. The PLS-DA model showed that the samples in THS group and control group are distributed in two separate areas, indicating a markedly different colonic metabolome (Figure 3B). The model parameters were *R*^2^*Y* = 0.907, *Q*^2^ = 0.105, which were very close to 1, thus indicating good ability of prediction and reliability of the model. In agreement with this result, OPLS-DA model also showed dramatic metabolic differences in THS and control rats (Figure 3C). Moreover, model validation by permutation test confirmed the reliability of PLS-DA model in explaining and predicting the variation between the two groups (Figure 3D).

**Figure 4:**
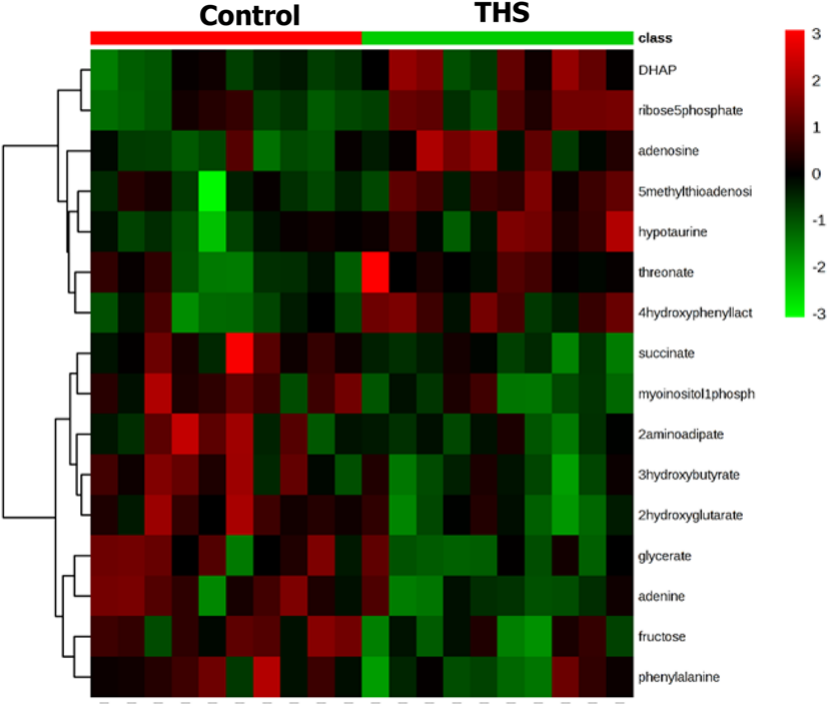
Heat map of significantly altered metabolites in colonic tissue of rats from control and THS groups

Heat map analysis showed that a total of 16 significantly altered metabolites with the value of variable importance in the VIP > 1 and *P*-value below 0.05 in PLS-DA model were identified to mainly contribute to the metabolic distinctions between THS and control rats (Figure 4). Metabolites that were significantly increased in THS-induced rats included dihydroxy acetone phosphate (DHAP) and ribose-5-phosphate, which are main products of the pentose phosphate pathway (Table 1). Moreover, metabolite associated with mitochondrial adenosine triphosphate (ATP) biosynthesis, such as adenosine, was also significantly increased in the colon of THS-induced rats (Table 1), whereas those that were significantly decreased included fructose and glyceric acid, which are critical biochemical metabolites involved in the metabolism of glycolysis (Table 2). Additionally, succinic acid, an important metabolic intermediate of tricarboxylic acid cycle (TCA), and myo-inositol-1-phosphate, 3-hydroxybutyric acid, 2-aminoadipic acid, phenylalanine, adenine, and 2-hydroxyglutaric acid, which mainly participated in the pathway of amino acid biosynthesis and metabolism, were also significantly decreased in the colon of THS-induced rats compared with that of control rats (Table 2).

**Table 1:** Significantly increased metabolites in the colon of THS-induced rats

Metabolites	VIP	*P*-value	Fold change
DHAP	2.45	3.29E-03	0.97
Ribose-5-phosphate	2.20	9.85E-03	0.60
Adenosine	2.24	8.65E-03	0.55
Threonic acid	2.05	1.82E-02	0.38
4-Hydroxyphenyllactic acid	2.60	1.42E-03	0.30
5-Methylthioadenosine	2.24	8.46E-03	0.26
Hypotaurine	2.19	1.07E-02	0.26

**Table 2:** Significantly decreased metabolites in the colon of THS-induced rats

Metabolites	VIP	*P*-value	Fold change
Succinic acid	2.39	4.37E-03	0.64
Fructose	2.32	6.03E-03	0.33
3-Hydroxybutyric acid	2.25	8.12E-03	0.31
Glyceric acid	2.04	1.90E-02	0.24
2-Aminoadipic acid	1.99	2.27E-02	0.25
Adenine	2.22	9.08E-03	0.25
2-Hydroxyglutaric acid	2.47	2.85E-03	0.20
myo-Inositol-1-phosphate	2.56	1.83E-03	0.19
Phenylalanine	1.87	3.30E-02	0.11

## Discussion

Understanding the mechanisms of underlying THS-induced intestinal barrier failure is of great importance for developing effective methods to treat THS-related disorders. The functional importance of the barrier function for the development of THS-induced MODS has been well documented in the small intestine [7–9]. However, little attention has been focused on the metabolic changes of the THS-induced intestinal injury and whether the barrier function of large intestine can also be affected by THS is also unknown. The current study showed that THS could induce severe damage to colonic barrier as evidenced by disrupted epithelial structure and loss of mucus layer in the colon. Furthermore, a significant increase in serum inflammatory factors was observed in THS-treated rats, indicating that THS-induced barrier failure might lead to the activation of systemic inflammatory responses. Importantly, we identified a series of metabolites in the injured colon of THS-treated rats, which might help explain the underlying mechanisms of THS-induced intestinal barrier dysfunction.

We first confirmed that the gut barrier failure could be induced by THS, which was consistent with the results of previous findings [8]. ZO-1 is an essential component of tight junction proteins that involved in the maintenance of intestinal barrier integrity [22]. Dysregulated expression of tight junction proteins has been demonstrated to be associated with the dysfunction of intestinal barrier [23]. In the present study, we demonstrated that the gut injury caused by THS may be relevant to the decreased expression of ZO-1. However, one limitation of the present study is that we did not examine the expression of other tight junction proteins such as Claudin and Occludin.

The gut consists of highly diverse microbes including bacteria and other microorganisms [24]. Mucus layer, which is formed by mucin protein secreted from intestinal goblet cells, is also an important component of intestinal barrier to limit the passage of bacterial-derived immune factors [25]. Indeed, the disruption of small intestinal mucus layer has been demonstrated to be associated with THS-induced gut barrier dysfunction [7,26]. Consistent with these findings, our result showed that colonic mucus layer was also disrupted as evidenced by the marked decrease in mucus-producing goblet cell numbers under the treatment of THS, although THS caused a significant increase in serum pro-inflammatory factors such as TNF-α, IFN-γ, IL-6, and IL-1β, which might be critical for the cause of chronic low-grade inflammation as well as the consequent initiation of MODS [27]. However, the level of microbial-derived lipopolysaccharide was not detected in the serum of THS-treated rats. Thus, further work was needed to confirm THS-induced MODS was mainly caused by which inflammatory factors.

**Figure 5:**
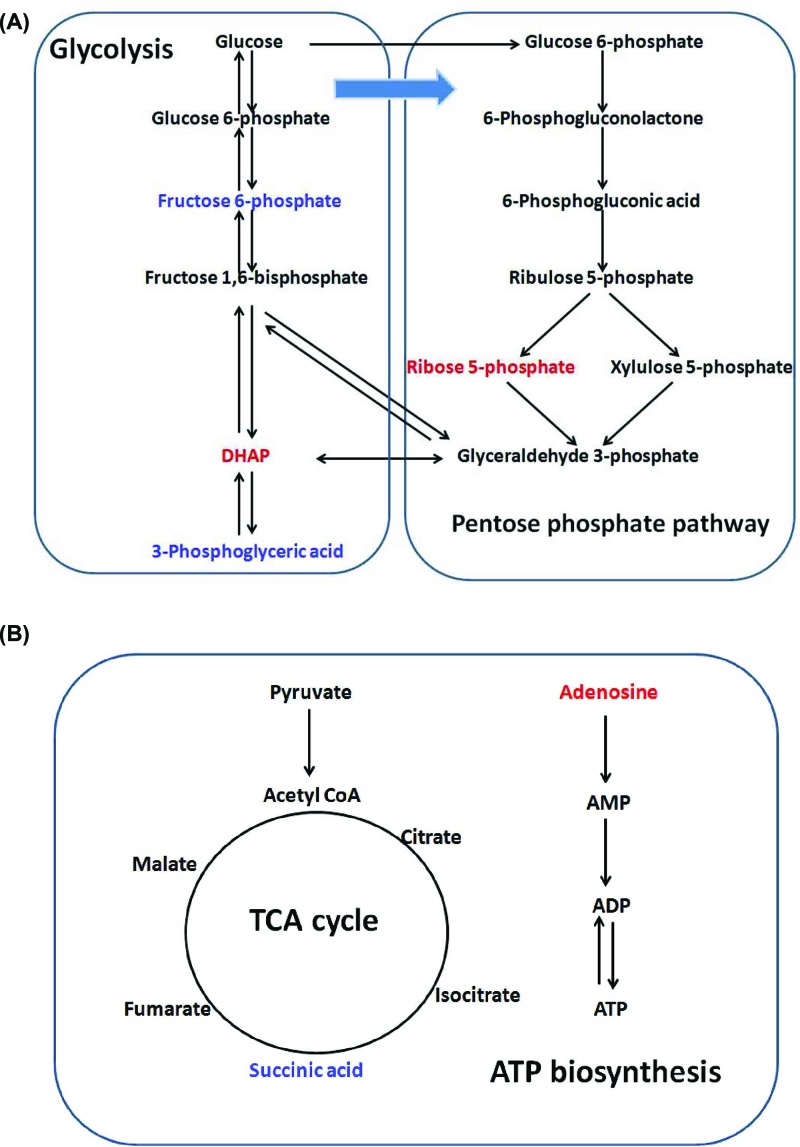
Network analysis of metabolic pathways in colonic tissue of THS-treated rats (**A**) Glycolysis and pentose phosphate pathway. (**B**) TCA cycle and ATP biosynthesis. Significantly increased metabolites in THS-treated rats are colored in red, whereas decreased metabolites in THS-treated rats are colored in blue. DHAP, dihydroxy acetone phosphate.

It has been reported that THS-induced injury can cause disrupted supply of oxygen and nutrients, which is the key factor for the induction of metabolic changes in specific tissues [12]. Thus, we hypothesized that THS might induce some metabolic changes to the intestine and the changed metabolites might provide some evidence to explain the mucus layer dysfunction caused by THS. Our metabolomics results showed that the levels of DHAP and ribose-5-phosphate were significantly increased in the colon of THS-treated rats compared with that of control rats. Ribose-5-phosphate is the key component for the synthesis of nucleotides and nucleic acids [26]. In the pentose phosphate pathway, it can be isomerized from ribulose 5-phosphate by action of the ribose-5-phosphate isomerase [29]. Consequently, glyceraldehyde 3-phosphate is formed from ribose 5-phosphate and xylulose 5-phosphate via the activity of transketolase [29]. It should be noted that glyceraldehyde 3-phosphate can also be isomerized to DHAP through the catalysis of triose phosphate isomerase [30]. Thus, the increased levels of DHAP and ribose-5-phosphate suggest that the pentose phosphate pathway might be promoted in response to THS-induced colonic injury (Figure 5A). The enhancement of pentose phosphate pathway has several significances and the main significance is that it can lead to the generation of reducing equivalents, such as NADPH, which will be used in reductive biosynthesis reactions within cells [31]. Moreover, higher quantities of ribose 5-phosphate and NADPH are needed for nucleotide and fatty acid synthesis during rapid cell growth [32]. Thus, we hypothesized that the promotion of pentose phosphate pathway caused by the induction of THS might initiate a protective mechanism to repair the damaged intestinal tract by promoting the growth and proliferation of intestinal epithelial cells. However, further mechanistic studies are required to confirm this notion.

Moreover, decreased levels of fructose and glyceric acid were also observed in the colon of THS-treated rats. Both of these metabolites are closely associated with the metabolism of glycolysis [33]. As stated above, DHAP isomerizes to the glyceraldehyde 3-phosphate and participates in the glycolytic pathway [28]. Therefore, the increased level of ribose 5-phosphate and decreased level of glyceric acid and fructose in the colonic tissue of THS-induced rats suggest that the intermediates such as DHAP in glycolysis can be diverted toward the pentose phosphate pathway under the induction of THS (Figure 5A). Succinic acid, which is generated in mitochondria via the TCA, is an important metabolic intermediate that is involved in multiple biological processes such as ATP biosynthesis and signaling transduction (Figure 5B) [34]. Dysregulated level of succinic acid usually happens in some genetic mitochondrial diseases, such as Leighs disease and Melas disease, and its degradation can lead to ATP synthesis dysfunction, malignant transformation, inflammation, and tissue injury (Figure 5B) [35]. Interestingly, an important substrate in ATP biosynthesis, adenosine, was found to be increased in the colonic tissue of THS-treated rats, suggesting that ATP biosynthesis may be promoted as a key mechanism in response to the intestinal injury caused by the THS. Thus, our data suggest that the decreased succinic acid and increased adenosine in the intestine may play an important role in contributing to the development of THS-induced intestinal barrier dysfunction. Whether supplementation of succinic acid or inhibition of adenosine may effectively prevent and control the severity of intestinal injury during the THS needs to be further investigated.

In conclusion, THS-induced metabolic changes of the colon in rats were characterized by the increased levels of DHAP, ribose-5-phosphate, and adenosine and decreased levels of fructose, glyceric acid, and succinic acid. The identification of these metabolites may help explain the pathogenesis of gut barrier failure or be used as potential metabolic biomarkers in THS-induced intestinal barrier dysfunction.

## Abbreviations

AB: alcian blue
ATP: adenosine triphosphate
DHAP: dihydroxy acetone phosphate
FITC: fluorescein isothiocyanate
GC: gas chromatography
H&E: hematoxylin and eosin
IFN: interferon
IL: interleukin
MODS: multiorgan dysfunction syndrome
MS: mass spectrometry
OPLS-DA: orthogonal partial least-squares discriminant analysis
PBS: phosphate-buffered saline
PLS-DA: partial least-squares discriminant analysis
TCA: tricarboxylic acid
THS: trauma-hemorrhagic shock
TIC: total ion current
TNF: tumor necrosis factor
VIP: Variable importance in the projection
ZO: zonula occludens
